# Regional decreases of cortical thickness in major depressive disorder and their correlation with illness duration: a case-control study

**DOI:** 10.3389/fpsyt.2024.1297204

**Published:** 2024-01-23

**Authors:** Fukun Wang, Xiaofang Hou, Xiao Guo, Chen Zang, Gang Wu, Jingjing Zhao

**Affiliations:** ^1^General Committee Office, Zhumadian Second People’s Hospital, Zhengzhou, Henan, China; ^2^Laboratory of Magnetic Resonance, Zhumadian Second People’s Hospital, Zhengzhou, Henan, China

**Keywords:** cortical thickness, major depressive disorder, left supramarginal gyrus, brain magnetic resonance imaging, T1

## Abstract

**Background:**

Alterations in brain structure and function in major depressive disorder (MDD) have been identified in a number of studies, but findings regarding cortical thickness were various and inconsistent. Our current study aims to explore the differences in cortical thickness between individuals with MDD and healthy controls (HC) in a Chinese population.

**Methods:**

We investigated T1-weighted brain magnetic resonance imaging data from 61 participants (31 MDD and 30 HC). The cortical thickness between the two groups and analyzed correlations between cortical thickness and demographic variables in the MDD group for regions with significant between-group differences were conducted.

**Results:**

Compared with the HC group, patients with MDD had significantly decreased cortical thickness, in left pars triangularis, left pars orbitalis, left rostral middle frontal gyrus, left supramarginal gyrus, right parahippocampal gyrus, right lingual gyrus, right fusiform and right inferior parietal gyrus. The cortical thickness of left rostral middle frontal gyrus was negatively correlated (*r* = −0.47, *p* = 0.028) with the illness duration in patients with MDD.

**Conclusion:**

Our study distinguished that cortical thickness decreases in numerous brain regions both in the left and right hemisphere in individuals with MDD, and the negative correlation between the cortical thickness of left rostral middle frontal gyrus illness duration. Our current findings are valuable in providing neural markers to identify MDD and understanding the potential pathophysiology of mood disorders.

## 1 Introduction

Major depressive disorder (MDD) is becoming the most burdensome mental disorder globally. The illness involves a depressed mood or loss of pleasure or interest in activities for long periods of time (1). Many alterations of behavioral symptoms are involved during the development process of MDD, including various fields related to emotions, motivation, cognition, and physiology (2). Although ongoing efforts to increase knowledge and skills for healthcare providers and clinical researchers, the pathogenies and pathophysiological processes of MDD are not fully understood (3). The existing evidence suggests that MDD may involve multiple levels of changes in genetics, biochemistry, imaging, and psychology. Due to neuroimaging research can reflect changes in the brain structure and function of MDD patients more intuitively, increasing studies are using this technology to explore the pathological mechanisms of MDD.

Cortical thickness refers to the component of gray matter volume, which is an index of cell density and health in the cerebral cortex (4). Thus, the alteration of cortical thickness represents an important signature for understanding emotional regulation of depression among all the neuroimaging approaches. For example, region-wise analysis reported that abnormal changes in the cortical thickness of the limbic system, such as the orbitofrontal lobe, cingulate gyrus, and other brain regions in untreated individuals with MDD, which may be related to abnormal emotional management and known as frontal limbic model of MDD (5). Temporal cortical thickness abnormalities were also reported in mood disorders (6). Two meta-analyses found that decreased cortical thickness in the orbitofrontal and temporal cortex of MDD (7, 8). A pilot study reported antidepressant treatment increased cortical thickness of the left medial OFC in adolescents with major depression (9). Several studies have also pointed out the increases in cortical thickness of specific brain regions in MDD. Qiu and colleagues reported cortical thickness in the right hemisphere in first-episode, treatment-naïve, mid-life MDD patients (10). Increased cortical thickness of several brain regions in the default mode network (DMN) of individuals with MDD was also reported in the meta-analysis (7). Specifically, Li et.al found that increased cortical thickness of posterior cingulate cortex, right ventromedial prefrontal cortex, and anterior cingulate cortex, and decreased cortical thickness in orbitofrontal cortex (gyrus rectus and orbital segment of superior frontal gyrus) and temporal cortex in medication-free patients with MDD.

Thus, to date, the existing studies related to cortical thickness in individuals with MDD are not well clarified. Results have been somewhat inconsistent across different studies. Our current study aims to explore the differences in cortical thickness of individuals with MDD and healthy controls in a Chinese population. According to the previous evidence, we hypothesize that the individuals with MDD will have thinner cortices in the frontal, temporal, limbic system, and parietal lobes (e.g., middle frontal gyrus and orbitofrontal cortex).

## 2 Methods

### 2.1 Ethical approval

The authors assert that all procedures contributing to this work comply with the ethical standards of the relevant national and institutional committees on human experimentation and with the Helsinki Declaration of 1975, as revised in 2008. All procedures involving human subjects/patients were approved by the Medical Ethics Committee of Zhumadian Second People’s Hospital in Henan Province (Approval no. IRB-2020-006-02). All participants provided written informed consent prior to participation.

### 2.2 Participants

All participants of this study were recruited from Zhumadian Second People’s Hospital in Henan Province. A total of 30 individuals diagnosed with MDD and 31 age and sex-matched healthy controls were included in the data analysis. All patients with MDD were recruited during a depressive episode, which were diagnosed by two professional and experienced psychiatrists. The inclusion criteria for MDD are as follows:(1) individuals meeting the diagnosis of major depressive disorder according to the Diagnostic and Statistical Manual of Mental Disorders, 5th edition(DSM-5); (2) Hamilton Depression Scale(HAMD)-24-item version scores≥20; (3) the patients taking medication were on a stable dose for at least 6 weeks or were unmedicated for at least 4 weeks; (4) 18–60 years old without gender not limited; and (5) primary school or above education level. The healthy controls had no history of mental illness or severe physical illness and no family history of mental illness. The exclusion criteria of all participants were as follows: (1) any history of neurological diseases, intellectual disability, other physical diseases, or comorbidities of other disorders; (2) any other mental disorders; (3) pregnancy or breastfeeding; and (4) head trauma resulting in loss of consciousness. The basic information of all participants can be seen in Table 1. There were only twenty-two patients with MDD having the illness duration, and eighteen patients having body mass index scores.

**Table 1:** Demographic information of participants.

Variable	MDD (*n* = 30)	HC (*n* = 31)	*p*-value
Age (years, mean ± SD)	35.67 ± 9.47	36.53 ± 9.21	0.720
Gender (female/male)	17/13	18/13	0.912
Illness duration (months, mean ± SD)	35.55 ± 47.81	–	–
Body mass index (kg/m^2^, mean ± SD)	22.63 ± 2.82	–	–

MDD, major depressive disorder; HC, healthy control; SD, standard deviation.

### 2.3 Image acquisition

The structural T1 images of all participants were scanned by using the 3D BRAVO with the following parameters: TR/ TE =6.77/2.49 ms, flip angle = 7o, matrix size = 256 × 256, voxel size = 1 × 1 × 1 mm3, 188 slices.

### 2.4 Preprocessing of T1 images

The T1 images were automatically preprocessed using the Computational Anatomy Toolbox version r1932.^1^ Briefly, the bias field correction was firstly performed for the T1 images, which were then segmented into gray matter, white matter and CSF. After removing brain stem and cerebellum, the cortical thickness was computed by using a projection scheme (11), which resulted in individual cortical thickness maps. This projection-based thickness estimation is fast and robust, which has been applied in other studies of neuropsychiatric disorders (12, 13). The individual maps of cortical thickness cannot be compared because they have a different number of vertexes. Thus, those maps were then warped and registered to standard space (fsaverage), thus, enabling matching of cortical locations among individuals across the whole surface. The registered cortical thickness maps were then smoothed with 12 mm full width at half maximum for statistical analysis.

### 2.5 Statistical analysis

The differences in gender and age between patients with MDD and HC were performed by using the chi-square test and two-sample *t*-test separately. The two-tailed two-sample *t*-test was also used to investigate the difference in cortical thickness at the vertex level between MDD patients and HC. The multiple comparisons were corrected using the false discovery rate (FDR) with *q* < 0.05.

If there were some brain areas that survived the FDR correction, mean cortical thickness of those brain areas was extracted for patients with MDD, and was used to compute the association with illness duration and body mass index by using Pearson correlation analysis. The statistical level of *p* < 0.05 was considered significant.

## 3 Results

The basic information of included participants is shown in Table 1. The average age of MDD group and HC group are 35.67 ± 9.47 years old and 36.53 ± 9.21, respectively. In total, seventeen females and thirteen males were included in the MDD group, and eighteen females and thirteen males were included in HC group. There was no significant difference (*p* > 0.05) in age and gender between patients with MDD and HC.

**Figure 1:**
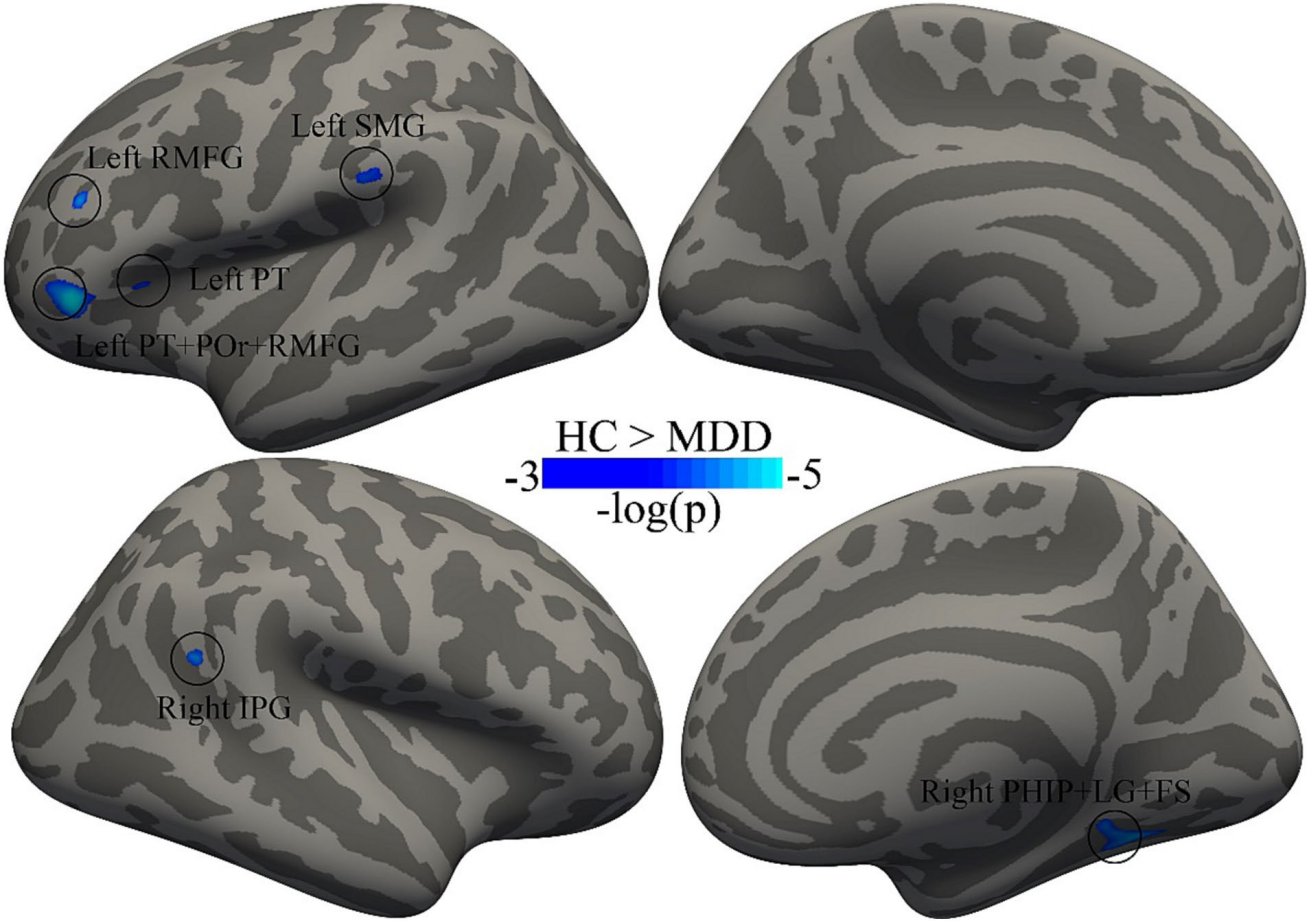
Decreased cortical thickness in patients with MDD compared with HC. The multiple comparisons were corrected using FDR with *q* < 0.05. MDD, major depressive disorder; HC, healthy controls; PT, pars triangularis; POr, pars orbitalis; RMFG, rostral middle frontal gyrus; SMG, supramarginal gyrus; PHIP, parahippocampal gyrus; LG, lingual gyrus; FS, fusiform; IPG, inferior parietal gyrus.

A two-sample *t*-test revealed that patients with MDD had significantly (FDR with *q* < 0.05) decreased cortical thickness, compared with HC, in left pars triangularis, left pars orbitalis, left rostral middle frontal gyrus, left supramarginal gyrus, right parahippocampal gyrus, right lingual gyrus, right fusiform and right inferior parietal gyrus (Figure 1 and Table 2). There were no brain areas showing increased cortical thickness in patients with MDD.

**Table 2:** Brain areas where the cortical thickness was significantly decreased in patients with MDD.

Brain regions	Number of vertex	Cluster size (mm^2^)	MNI coordinate			*t*-value	Effect size (Cohen’s d)
			*X*	*Y*	*Z*		
Left PT+ POr + RMFG	390	231.09	−47	36	−4	−4.76	1.22
Left RMFG	59	32.08	−41	35	22	−2.43	0.62
Left SMG	94	41.83	−61	−28	22	−4.16	1.07
Left PT	35	12.40	−34	26	8	−4.1	1.05
Right PHIP+ LG+ FS	394	209.2	33	−56	−8	−4.51	1.16
Right IPG	73	29.41	50	−47	22	−4.36	1.12

MDD, major depressive disorder; PT, pars triangularis; POr, pars orbitalis; RMFG, rostral middle frontal gyrus; SMG, supramarginal gyrus; PHIP, parahippocampal gyrus; LG, lingual gyrus; FS, fusiform; IPG, inferior parietal gyrus.

**Figure 2:**
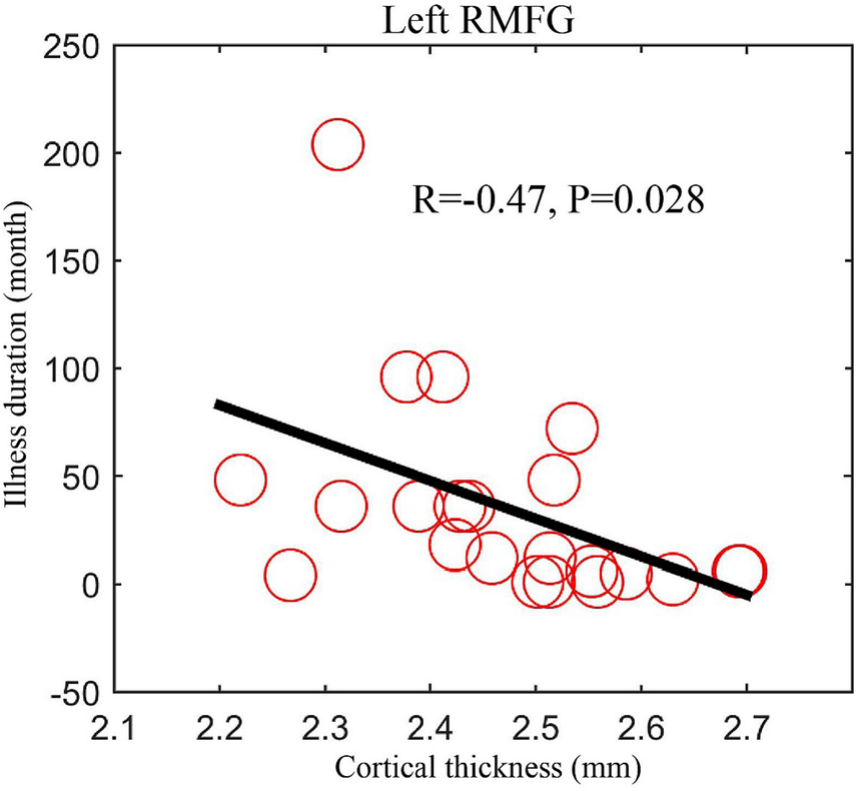
The negative correlation between cortical thickness of left RMFG and illness duration in patients with MDD. MDD, major depressive disorder; RMFG, rostral middle frontal gyrus.

In addition, we found that the cortical thickness of left rostral middle frontal gyrus was negatively correlated (*r* = −0.47, *p* = 0.028) with the illness duration in patients with MDD (Figure 2). We conducted a sensitivity analysis to explore the relationship between the course of the disease and cortical thickness after excluding a value of very long illness duration, and the results showed that the difference was still statistically significant (*r* = −0.43, *p* = 0.047, Supplementary Figure S1). We did not find significant correlation between cortical thickness of those brain areas and body mass index.

## 4 Discussion

By utilizing T1 weighted anatomical magnetic resonance imaging (MRI) images, we investigated the changes in cortical thickness in individuals with MDD. The main findings of current study are as follows: (1) four left hemisphere brain regions (i.e., pars triangularis, pars orbitalis, rostral middle frontal gyrus, and supramarginal gyrus) were found to have thinner cortical thickness in individuals with MDD when compared to HC; (2) the decreases in cortical thickness of three right hemisphere brain regions (i.e., parahippocampal gyrus, lingual gyrus, fusiform, and inferior parietal gyrus) was also reported in MDD; and (3) the cortical thickness of left rostral middle frontal gyrus was negatively correlated with the illness duration in individuals with MDD. The results reported in our study provided new evidence for exploring alterations in the brain structure of MDD.

Consistent with the abnormal cortical thickness observed in previous studies, we observed a decrease in cortical thickness in several regions of the left hemisphere of the brain. It is worth noting that we have found that left rostral middle frontal gyrus was negatively correlated with the illness duration in individuals with MDD. The rostral middle frontal gyrus is partly located in dorsolateral prefrontal cortex and the control network of brain (14), and it plays an important role in dysfunctional emotional processing, frontal executive function, working memory, and problem solving (14). Consistent with our results, a study focused on the thickness and depression reported that the cortical thickness of rostral middle frontal gyrus was negatively related to positive emotions at small effect sizes (accounting for 0.2–2.4% of variance; p-fdr: 0.0051–0.1900) (15). Song and colleagues reported that left rostral middle frontal gyrus thickness was negatively correlated with genetic risk score at 0.05 threshold (corrected *p* < 0.05), and mediates the relationship between genetic risk and neuroticism traits (16). Another study focused on the brain imaging of bipolar disorder also found significantly thinned left rostral middle frontal gyrus in individuals with patients when compared with the healthy controls (*d* = −0.276; *p* = 2.99 × 10^−19^) (17). Our findings and the above evidence suggested that left rostral middle frontal gyrus is a potential hallmark to distinguish mood disorders, and may be negatively correlated to the positive emotions, genetic risk score and illness duration of depression. However, some studies have proposed inconsistent views. Qiu et al. and *van* Eijndhoven et al. reported increased cortical thickness in right rostral middle frontal gyrus in first-episode, medication-free MDD patients (5, 10). Reynolds and colleagues found both right and left rostral middle frontal gyrus were thicker in youth with MDD than in controls (*p* = 0.009; Left – controls: 2.74 ± 0.28, MDD: 2.94 ± 0.25; Right – controls: 2.77 ± 0.26, MDD: 2.80 ± 0.28) (18). Thus, characteristics of rostral middle frontal gyrus in MDD patients can be explored through meta-analysis, and it is also worthy of further confirmation by large samples.

Left pars triangularis is located in ventrolateral prefrontal cortex, and it has been reported to be related to cognitive control (19). Consistent with our findings, a case–control study also reported thinner cortical thickness of left pars triangularis in MDD group when compared with HC group (20). Functional MRI data analysis with a semantic task indicated that left inferior frontal cortex (pars triangularis) contributed to the classification of depression and controls (21).

We also found a joint region of left pars triangularis+ pars orbitalis+rostral middle frontal gyrus had a decrease in cortical thickness in individuals with MDD. Similar to pars triangularis, pars orbitalis also plays important roles in the language production network (22). We did not find much evidence to focus on changes in the pars orbitalis brain region and its function in MDD. A brain structure study with children and adolescents suggests that it may be higher impulsivity, but not depressive symptoms, was associated with reduced cortical thickness in the pars orbitalis (23). Moreover, the cortical thickness of left supramarginal gyrus was inconsistently reported in previous studies (4, 10, 24, 25). The potential roles of left supramarginal gyrus in MDD also need to be further clarified.

Regarding the brain regions mentioned in the right hemisphere with cortical thickness decreases in our results, to the best of our knowledge, previous studies have focused more on exploring changes in their functional connections. For example, a case–control study reported late-life depression exhibited lower intrinsic functional connectivity in right inferior parietal gyrus and other right fronto-parietal network (FPN) (26), However, another study included 25 patients with recurrent depression found functional connectivity was considerably decreased in right inferior parietal gyrus after 8 weeks treatment (27). Few studies have pointed out the causes and rules of the structural and functional changes of right inferior parietal gyrus in patients with depression. The existing evidence suggests that right inferior parietal gyrus might be a crucial hub in transferring information between these abnormal regions (26).

Our results also reported a decrease in cortical thickness of the occipito-temporal cluster (i.e., right parahippocampal gyrus+ lingual gyrus+ fusiform) in MDD. Similarly, a study with an overlapping twin and sibling sample reported the reduction of surface area in an occipito-temporal cluster, which comprised part of the right lingual, fusiform and parahippocampal gyri (28). The decrease of cortical thickness the right fusiform in MDD cases with comorbid generalized anxiety were also reported previously (29). The meta-analysis by the ENIGMA-MDD group also found a significant reduction of right lingual gyrus surface area, but nonsignificant association for fusiform or parahippocampal, in adolescent depression (30). Previous evidence pointed out the reduced cortical thickness of occipito-temporal cluster may be associated with visual memory and attention deficits in depression (31). The right lingual gyrus may be associated with cognitive functions in MDD. The evidence from ENIGMA-MDD group and other studies points to differences in orbitofrontal and cingulate cortexes between MDD and healthy controls (30, 32). However, our study did not provide such evidence, which is not surprising. This may be due to the significant heterogeneity in both clinical manifestations and brain structure among patients with MDD. The underlying reasons for the structural and functional alterations of these brain regions deserve further exploration.

The current study demonstrated a decrease in cortical thickness in several brain regions of individuals with MDD in a Chinese population, which provides new evidence for the neuroimaging approaches to mood disorders. However, several limitations should be noted in the present psychiatric neuroimaging study. Firstly, our study is based on a single institutional database, a certain degree of selection bias may limit our extrapolation of results. Secondly, the information on BMI was only collected in MDD group, while the medication records of patients were not available from our collected data. The evidence from ENIGMA-MDD group reported obesity (BMI > 30) was significantly associated with both mass univariate and multivariate pattern recognition analyses independent of MDD diagnostics (33). Their results suggested a neurobiological interaction between obesity and brain structure under physiological and pathological brain conditions. Thus, obesity may affects the brain just as much as a neuropsychiatric condition would and should be treated taking this into account. However, our study did not find association between BMI and with brain cortical thickness in MDD. We think this may be related to our small sample size and limited number of overweight and obese individuals in current study. In our study sample, only 8 out of all 30 depressed patients met the criteria for overweight, and no study subjects met the criteria for obesity. Since the BMI of our study subjects is generally within the normal range, it may require more individuals with ultra-high BMI to determine alterations in brain cortical thickness. Thirdly, our study is a cross-sectional study, so we cannot determine whether the brain structure of MDD undergoes changes after treatment.

Moreover, our sample consists of first-episode and recurrent patients, we cannot exclude the potential influence of the previous treatment effects and their influence on reported findings. Additionally, we did not control for other information, such as maternal status, professional activity, and manual laterality, and we also did not control that the research subject must be right-handed. These variables should be considered in future studies. Many residue confounders might as well affect the findings of the study. Larger samples and longitudinal research are needed to explore whether the decrease in cortical thickness in MDD patients can be improved through drug treatment in the future.

## 5 Conclusion

Our findings serve as a supplement to the evidence of alterations in cortical thickness among individuals with MDD in the Chinese population. In summary, our study distinguished that cortical thickness decreases in numerous brain regions (i.e., pars triangularis, pars orbitalis, rostral middle frontal gyrus, and supramarginal gyrus of the left hemisphere; and parahippocampal gyrus, lingual gyrus, fusiform, and inferior parietal gyrus of the right hemisphere) in individuals with MDD. Moreover, the cortical thickness of left rostral middle frontal gyrus was negatively correlated with the illness duration of the disorder. Our current findings are valuable in providing neural markers to identify MDD, which contribute to the clinical diagnosis of affective disorders and further improve our understanding of the potential pathophysiology of MDD.

## Data availability statement

The raw data supporting the conclusions of this article will be made available by the authors, without undue reservation.

## Ethics statement

The studies involving humans were approved by Medical Ethics Committee of Zhumadian Second People’s Hospital. The studies were conducted in accordance with the local legislation and institutional requirements. Written informed consent for participation in this study was provided by the participants’ legal guardians/next of kin. Written informed consent was obtained from the individual(s) for the publication of any potentially identifiable images or data included in this article.

## Author contributions

CZ: Formal analysis, Investigation, Methodology, Writing – original draft. BM: Formal analysis, Writing – original draft, Software. XH: Writing – original draft, Methodology, Writing – review & editing. XG: Writing – review & editing, Supervision. FW: Writing – review & editing, Conceptualization. HG: Supervision, Writing – review & editing. JZ: Writing – review & editing.

## Footnotes

^1^ https://neuro-jena.github.io/cat/
