# Supplementary material for: Regional decreases of cortical thickness in major depressive disorder and their correlation with illness duration: a case-control study
Source: Front Psychiatry. 2024 Jan 23;15:1297204. doi: 10.3389/fpsyt.2024.1297204 (PMC10844537; doi:10.3389/fpsyt.2024.1297204)
Supplement: Supplementary file 1 [file Table_1.DOCX]

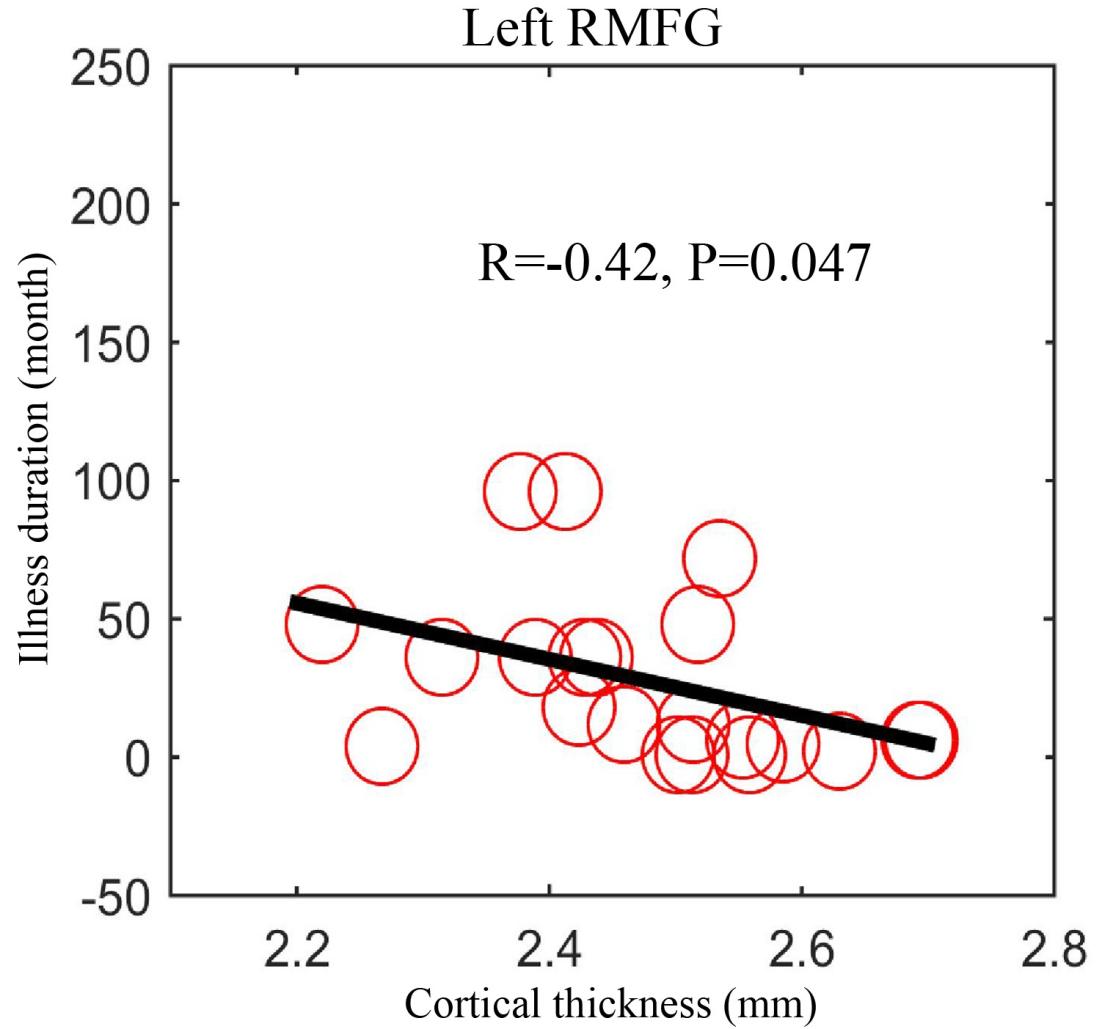


**Supplemental Figure 1.** The negative correlation between cortical thickness of left RMFG and illness duration in patients with MDD after excluding a value of very long illness duration. MDD: major depressive disorder; RMFG: rostral middle frontal gyrus.
